# Supplementary material for: Clinical Value of Inflammatory and Neurotrophic Biomarkers in Bipolar Disorder: A Systematic Review and Meta-Analysis
Source: Biomedicines. 2022 Jun 9;10(6):1368. doi: 10.3390/biomedicines10061368 (PMC9220136; doi:10.3390/biomedicines10061368)
Supplement: Supplementary file 1 [file biomedicines-10-01368-s001.zip › biomedicines-1714169-supplementary.pdf]

Table S1. Supplementary information on the 51 studies included in the present systematic review and meta-analysis.

ID	First author-year	Complete sample	Mean age (range)	Sex (M/F)	Study design	Diagnosis	Assay	Blood fraction	Medication	Use/abuse substances	Financing
1.	Bai et al. (2014)	130 BD patients (77 BD-I, 53 BD-II) (75 euthymic, 14 (hypo)manic state, 41 depressive state)<br>130 Healthy controls	44.55±11.08 BD (47.0±11.7 BD-I, 41.0±10.2 BD-II) (44.9±12.4 euthymic, 46.1±9.3 (hypo)manic state, 43.4±10.2 depressive state)<br>41.8±9.7 HC	31.55%/68.44% BD (33.8%/66.2% BD-I, 28.3%/71.7% BD-II) (33.3%/66.7% euthymic, 21.4%/78.6% (hypo)manic state, 33.3%/66.7% depressive state)<br>35.4%/64.6% HC	Cross-sectional study	MINI DSM-IV diagnosis criteria	ELISA	Serum	Drug-naive and medicated	Excluded	Yes
2.	Barbosa et al. (2010)	53 BD-I patients (34 mania, 19 euthymia)<br>38 Healthy controls	47.77±13.01 BD-I (49.6±14.2 mania, 44.5±10.9 euthymia)<br>42.9±9.7 HC	39.59%/60.40% BD-I (38.2%/61.8% mania, 42.1%/57.9% euthymia)<br>47.4%/52.6% HC	Cross-sectional study	MINI-plus	ELISA	Plasma	Yes	Included	Yes
3.	Barbosa et al. (2011)	49 BD-I patients (30 manic, 19 euthymic)<br>26 Healthy controls	46.85±12.56 BD-I (48.03±13.66 manic, 45.00±10.84 euthymic)<br>42.94±9.39 HC	40.81%/59.18% BD-I (40.00%/60.00% manic, 42.1%/57.90% euthymic)<br>47.2%/52.80% HC	Cross-sectional study	MINI-plus	sandwich-ELISA	Plasma	Yes	Not specified	Yes
4.	Barbosa et al. (2012)	25 BD-I euthymic patients<br>25 Healthy controls	50.88±9.11 BD-I<br>48.04±7.08 HC	32%/68% BD-I<br>44%/56% HC	Cross-sectional study	MINI	ELISA	Plasma	Yes	Not specified	Yes
5.	Barbosa et al. (2014)	46 BD-I patients (23 euthymic, 23 manic)<br>23 Healthy controls	49.74±11.41 BD-I (50.39±8.07 euthymic, 49.09±14.76 manic)<br>50.04±6.31 HC	26.09%/73.91% BD-I (17.39%/82.61% euthymic, 34.79%/65.21% manic)<br>21.74%/78.26% HC	Cross-sectional study	MINI-Plus	ELISA	Plasma	Yes	Not specified	Yes
6.	Benedetti et al. (2017)	40 BD-I inpatients current major depressive episode	46.3±13.85 BD-I	32.5%/67.5% BD-I	Cross-sectional study	Clinical interview	ELISA	Serum	Not specified	Excluded	Yes
7.	Bonnin at al. (2019)	102 BD patients (47 psychoeducation [25 BD-I], 39 FR [40 BD-I], 16 TAU [14 BD-I])	40.95±8.20 BD (35.2±9.2 psychoeducation, 48.5±6.3 FR, 39.5±9.9 TAU)	63.20%36.79% BD (53.2%/46.8% psychoeducation, 73%/27% FR, 68.7%/31.3% TAU)	Naturalistic cohort study	SCID	ELISA	Serum	Excluded	Excluded	Yes
8.	Brietzke et al. (2009)	61 BD-I patients (14 euthymic, 23 manic episode, 24 depressive episode)<br>25 Healthy controls	42.23±13.00 BD-I (44.2±13.75 euthymic, 40.8±13.70 manic, 45.0±11.91 depressive)<br>43.4±12.25 HC	39.33%/60.65% BD-I (28.6%/71.4% euthymic, 47.8%/ 52.2% manic, 37.5%/62.5% depressive)<br>20.0%/80.0% HC	Cross-sectional study	SCID-I	CBA	Serum	Not specified	Excluded	No
9.	Cetin et al. (2012)	45 BD-I patients in euthymic state (22 subsyndromal symptoms, 23 without subsyndromal symptoms)<br>23 Healthy controls	35.71±7.15 BD-I (36.86±7.03 subsyndromal, 34.61±7.28 without subsyndromal)<br>31.65±5.21 HC	44.47%/55.52% BD-I (45.5%/54.5% subsyndromal, 43.5%/56.5% without subsyndromal)<br>47.8%/52.2% HC	Cross-sectional study	Clinical interview SKIP-TURK	ELISA	Plasma	Yes	Excluded	Yes

10.	Chang et al. (2017)	91 BD patients<br>75 Healthy controls	43.0±12.2 BD<br>44.03±16.43 HC	42.9%/57.1% BD<br>41.3%/58.7% HC	Cross-sectional study	DIGS	ELISA	Plasma	Yes	Excluded	Yes
11.	Chiou & Huang (2019)	83 BD-I patients (61 mania, 22 depression)<br>222 Healthy controls	36.8±11.4 BD-I<br>34.3±7.0 HC	45.78%/54.21% BD-I<br>36.03%/63.96% HC	Cross-sectional study	SCID-I	ELISA	Serum	Off medication for at least one week	Excluded	Yes
12.	Dell'Osso et al. (2010)	33 BD outpatients current major depressive episode (17 unipolar, 16 BD-I)<br>15 Healthy controls	46.4±14.3 (22-65 years) BD (46.06±10.78 unipolar, 46.75±17.67 BD-I)<br>46.9±9.2 HC	36.37%/63.63% BD (23.5%/76.5% unipolar, 50%/50% BD-I)<br>20%/80% HC	Cross-sectional study	MINI	ELISA	Plasma	Drug-naïve (n=6), drug-free at least for 2 weeks (n=9) and medicated with different combinations of drugs (n=18)	Excluded	Not specified
13.	Dolsen et al. (2018)	22 BD-I euthymic patients (11 CBT for insomnia, 11 psychoeducation)	36,4±11.85 BD-I	54,5%/45,5% BD-I	Post-hoc analysis of a clinical trial	SCID-I	ELISA	Saliva	Yes	Not specified	
14.	Du et al. (2017)	48 BD patients (typical BD group)<br>63 BD with anxiety disorders patients (atypical BD group)<br>111 Anxiety disorders patients	33.06±14.53 BD<br>34.33±15.55 atypical BD<br>37.38±12.15 anxiety	31.3%/68.8% BD<br>28.6%/71.4% atypical BD<br>35.1%/64.9% anxiety	Retrospective and prospective study	ICD-10 diagnostic criteria for bipolar disorder	Not specified	Not specified	Yes	Included	Not applicable
15.	Grande et al. (2014)	115 BD patients (79 BD-I, 18 BD-II, 18 NOS)<br>25 First-degree relatives	44.0±20.0 (18-65 years) BD<br>41.0±32.5 First-degree relatives	29.6%/70.4% BD<br>24.0%/76.0% First-degree relatives	Historical cohort study	SCID-I	ELISA	Serum	Not specified	Not specified	Yes
16.	Hope et al. (2015)	111 BD patients (65 BD-I, 40 BD-II, 6 NOS)<br>121 SCZ patients<br>241 Healthy controls	33±10 (18-63 years) BD<br>36±10 SCZ<br>36±12 HC	54%/46% BD<br>45%/55% SCZ<br>39%/61% HC	Cross-sectional study	SCID-I	EIAs	Plasma	Yes	Included	Yes
17.	Isgren et al. (2015)	121 BD patients (65 BD-I, 46 BD-II, 10 NOS)<br>71 Healthy controls	36.0±22.0 BD<br>32.0±16.0 HC	38.8%/61.2% BD<br>36.6%/63.4% HC	Cross-sectional study	ADE	MSD 96-well multi-array and multi-spot human cytokine assay (Human Cytokine Assay Ultra-Sensitive kit)	Serum	Yes	Included	Yes
18.	Jacoby et al. (2016)	60 BD-I patients hospitalized with a manic or mixed episode<br>35 Healthy controls	42.7±11.7 (20–60 years) BD-I<br>36.7±11.6 (18–59 years) HC	61.7%/38.3% BD-I<br>57.1%/42.9% HC	Prospective study (6-12 months follow-up)	SCAN ICD-10 diagnosis criteria	ELISA	Plasma	Yes	Excluded	Yes
19.	Karabulut et al. (2019)	107 BD patients (77 BD chronic [50 euthymic state, 20 manic state, 7 depressive state], 30 BD early-stage	34.29±10.24 BD (37.81±11.9 BD chronic, 25.27±6 BD early-stage)<br>31.7±11.8 HC	52.34%/47.65% BD (57.2%/42.8% BD chronic, 23.4%/76.6% BD early-stage)<br>56.7%/43.3% HC	Cross-sectional study	SCID-I	ELISA	Plasma	Yes	Excluded	Not specified

		patients [12 euthymic state, 13 manic state, 2 depressive state, 2 hypomanic state, 1 mixed state])<br>30 Healthy controls								
20.	Kauer-Sant'Anna et al. (2007)	163 BD-I and BD-II patients (78 with life-time exposure to a traumatic event, 85 without-lifetime exposure to a traumatic event)	42.5 ±11.58 BD (42.13±12.00 presence of trauma, 43.01±11.21 absence of trauma)	28.18%/71.82% BD (25.0%/75% presence of trauma, 31.1%/68.9% absence of trauma)	Cross-sectional study	SCID-I	ELISA	Serum	Yes	Included	Yes
21.	Kenna et al. (2014)	47 BD euthymic patients (13 BD-I, 20 BD-II, 14 NOS)<br>26 Healthy controls	32.97±6.5 (18-45 years) BD (32.9±6.4 BD-I, 32.1±6.3 BD-II, 34.3±6.9 NOS)<br>31.8±6.4 HC	0%/100% BD<br>0%/100% HC	Cross-sectional study	SCID	ELISA	Plasma	70% stable pharmacological treatment/30% no treatment	Excluded	Yes
22.	Kim et al. (2013)	116 BD-I patients in manic episode, with pharmacological treatment during 6 weeks of follow-up<br>123 Healthy controls	35.90±11.76 (35.99±11.67) BD-I<br>35.45±10.37 HC	36.2%/63.8% (37.3%/62.7%) BD-I<br>45.53%/54.47% HC	Longitudinal study (6 weeks)	SCID-I	ELISA	Plasma	Medicated, drug-naive and drug-free at baseline; pharmacological treatment during 6 weeks of follow-up	Not specified	Yes
23.	Lee et al. (2014)	232 BD-II patients in depressed state, with VPA treatment during 12 weeks of follow-up<br>(115 VPA+Memantine, 117 VPA+placebo)	31.77±11.56 BD-II	50.86%/49.13% BD-II	Clinical trial (6-week, double-blind)	SADS-L, Chinese version	Antibody pair assay system	Plasma	Medicated at baseline (VPA); low-dose memantine or placebo during 12 weeks of follow-up with randomization	Excluded	Yes
24.	Lee et al. (2016)	541 BD patients (117 BD-I, 424 BD-II), with add-on memantine/placebo/ dextromethorphan treatment during 12 weeks of follow-up	31.2±11.2 BD	48.79%/51.20% BD	Post-hoc analysis of two clinical trials	SADS-L, Chinese Version	ELISA	Plasma	Medicated at baseline (VPA); add-on memantine/placebo/ dextromethorphan treatment during 12 week of follow-up	Excluded	Not specified
25.	Loch et al. (2015)	23 BD patients in depressive episode (8 BD-I, 15 BD-II), with litium tratment during 6 weeks of follow-up<br>28 Healthy controls	28.0 (18-43 years) BD<br>28.3 (17-51 years) HC	17.4%/82.6% BD<br>46.4%/53.6% HC	Longitudinal study (6 weeks)	SCID	ELISA	Plasma	Drug-naive and drug-free al baseline; lithium treatment during 6 weeks of follow-up	Excluded	Yes
26.	Machado-Vieira et al. (2007)	30 BD-I patients in mania state (22 drug-naïve, 8 drug-free)<br>30 Healthy controls	26±4 (20-40 years) BD-I<br>26.5±5.2 HC	23.3%/76.7% BD-I<br>23.3%/76.7% HC	Cross-sectional study	SCID-I	ELISA	Plasma	Drug-naïve and drug-free	Not specified	Yes
27.	Maiti et al. (2017)	25 BD patients current episode mania, 21 with oxcarbazepine treatment during 4 weeks of follow-up<br>25 Healthy controls	34.16±9.89 (18-45 years) BD<br>34.44±9.53 HC	80%/20% BD<br>80%/20% HC	Prospective, interventional, open label clinical study (4 weeks)	SCAN	ELISA	Serum	Not specified at baseline; oxcarbazepine treatment during 4 weeks of follow-up	Excluded	Not specified

28.	Mansur et al. (2020)	55 BD I-II patients current major depressive episode (27 Infliximab + 28 placebo)	BD 44.91 ± 10.90 (44.04 ± 11.55 Infliximab, 45.75 ± 10.28 placebo)	20%/80% BD (25.9%/74.1% Infliximab, 14.3%/85.7% placebo)	Clinical trial (12-week, double-blind)	MINI 5.0.0 for the DSM-IV-TR	Multiplex assay/ECLIA	Plasma	Medicated at baseline; pharmacological treatment during 12 weeks of follow-up	Not specified	Yes
29.	Mizuno et al. (2016)	60 BD patients<br>60 SCZ patients<br>60 Healthy controls	50.2±13.8 (19–75 years) BD<br>45.9±10.0 (25–70 years) SCZ<br>41.0±17.6 (18–77 years) HC	46.7%/53.3% BD<br>36.7%/63.3% SCZ<br>50.0%/50.0% HC	Cross-sectional study	MINI	ELISA	Plasma	Yes	Included	Yes
30a.	Mondin et al. (2016)	48 BD patients (10 mania/mixed episode, 27 depressive episode, 11 euthymic) 73 MDD<br>94 Healthy controls	21.92 ± 2.32 (18-24 years) BD<br>21.86 ± 2.05 MDD<br>22.40 ± 2.25 HC	25%/75% BD<br>24.7%/75.3% MDD<br>42.6%/57.4% HC	Cross-sectional study	SCID-I<br>MINI	ELISA	Serum	Drug-naïve	Not specified	Yes
30b.	Wiener et al. (2017)	48 BD patients<br>48 MDD patients<br>48 Healthy controls	21.92±2.32 (18-24 years) BD<br>21.81±2.14 MDD<br>21.88±2.31 (18-24 years) HC	25%/75% BD<br>25%/75% MDD<br>25%/75% HC	Cross-sectional study	SCID-I<br>MINI	ELISA	Serum	Drug-free	Not specified	Not specified
31.	Monteleone et al. (2008)	28 BD euthymic patients (17 BD-I, 11 BD-II)<br>35 UD patients (24 euthymic, 11 MDE)<br>22 Healthy controls	45.10±11.08 BD (46.6±9.4 BD-I, 42.8±13.7 BD-II)<br>49.2±12.7 UD euthymic,<br>45.7±13.6 UD MDE<br>40.1±16.4 HC	39.28%/60.70% BD (29.41%/70.58% BD-I, 54.54%/45.45% BD-II)<br>20.83%/79.16% UD euthymic,<br>18.18%/81.81% UD MDE<br>36.36%/63.63% HC	Cross-sectional study	SCID-IP, Patient Edition	ELISA	Serum	Medicated and drug-free	Not specified	Yes
32.	Mora et al. (2019)	84 BD patients (52 euthymic, 32 manic)<br>49 Healthy controls	45.13±12.28 (18-65 years) BD (47.52±11.9 euthymic BD, 41.25±12.9 manic)<br>48.3±12.1 HC	52.38%/47.62% BD (50%/50% euthymic, 56.3%/43.7% manic)<br>42.9%/57.1% HC	Cross-sectional study	SCID-I	ELISA	Serum	Yes	Excluded	Yes
33a.	Munkholm et al. (2014)	37 BD rapid cycling patients (22 BD-I, 15 BD-II)<br>40 Healthy controls	40.9±12.3 (18-70 years) BD<br>36.3±12.5 HC	32.43%/67.56% BD<br>42.5%/57.5% HC	Longitudinal study	SCAN<br>HCl-32	ELISA	Plasma	Yes	Excluded	Yes
33b.	Munkholm et al. (2015)	37 BD rapid cycling patients (22 BD-I, 15 BD-II)<br>40 Healthy controls	40.9±12.3 (18-70 years) BD<br>36.3±12.5 HC	32.43%/67.56% BD<br>42.5%/57.5% HC	Longitudinal study	SCAN<br>HCl-32	ELISA	Plasma	Yes	Excluded	Yes
34.	Ortiz-Dominguez et al. (2007)	20 BD-I patients (10 manic episode, 10 depressive episode)<br>33 Healthy controls	34.3±9.94 (20-50 years) BD-I (28.9±8.45 manic, 39.7±11.43 depressive)<br>32.3±10.8 HC	25%/75% BD-I (30%/70% manic, 20%/80% depressive)<br>15.15%/84.84% HC	Cross-sectional study	MINI for DSM-IV	ELISA	Not specified	Drug-naive and drug-free	Excluded	Yes
35.	Pantović-Stefanović et al. (2016)	83 BD-I patients, with pharmacological treatment during 10 weeks of follow-up<br>73 Healthy controls	45.61±11.05 BD-I<br>45.82±8.19 HC	36.4%/63.60% BD-I<br>43.2%/56.8% HC	Longitudinal study (6 weeks)	SCID-I	ELISA	Serum	Unmedicated at baseline; pharmacological treatment during 10 weeks of follow-up	Excluded	Yes
36.	Quidé et al. (2018)	69 BD-I patients<br>68 SCZ patients<br>72 Healthy controls	38.11±12.31 (18-65 years) BD-I<br>41.78±11.33 SCZ<br>36.17±11.57 HC	33.33%/66.66% BD-I<br>57.35%/42.64% SCZ<br>52.77%/47.22% HC	Cross-sectional study	OPCRIT algorithm applied to interviewer ratings on the Diagnostic Interview for	ELISA	Serum	Yes	Excluded	Yes

						Psychosis criteria					
37.	Reininghaus et al. (2015)	101 BD euthymic patients (64 BD-I, 37 BD-II) (35 WCYC, 66 non-WCYC)<br>48 Healthy controls	44.00±12.99 BD (39.1±11.1 BD WCYC, 46.6±14.0 BD non-WCYC)	48.51%/51.48% BD<br>41.66%/58.33% HC	Cross-sectional study	SCID-I	ECLIA	Serum	Yes	Not specified	Yes
38.	Rosa et al. (2006)	44 BD-I patients (15 manic, 14 depressed, 15 euthymic)<br>14 Healthy controls	40.83±9.29 (30-51 years) BD-I (40.1±9.3 manic, 42.1±8.2 depressed, 40.4±10.3 euthymic)<br>41.1±11.1 HC	41.07%/58.95% BD-I (56.3%/43.8% manic, 28.6%/71.4% depressed, 37.5%/62.5% euthymic)<br>31.2%/68.8% HC	Cross-sectional study?	SCID-I	Western blot analysis	Serum	Yes	Not specified	Yes
39a.	Siwek et al. (2016)	133 BD patients (65 BD-I and 64 BD-II) (23 manic phase, 61 depressive phase, 49 euthymic) (35 with melancholia, 26 without melancholia)<br>50 Heathy controls	44.3±12.9 (21-70 years) BD<br>45.8±12.4 HC	65.41%/34.58% BD<br>72%/28% HC	Case-control study	SCID-I	ELISA	Serum	Yes	Included	Yes
39b.	Sowa-Kućma et al. (2017)	133 BD patients (69 BD-I, 64 BD-II all in depressed or euthymic phase)<br>114 MDD patients<br>50 Healthy controls	44.3±12.9 BD (42.0 ± 14.6 BD-I, 46.8±10.2 BD-II)<br>49.4±10.7 MDD<br>45.8±12.4 HC	65.41%/34.58% BD (42.02%/57.97% BD-I, 26.56%/73.43% BD-II)<br>35.96%/64.03% MDD<br>28%/72% HC	Case-control study	SCID-I	ELISA	Serum	Not specified	Excluded	Yes
40.	Soares et al. (2016)	118 BD patients in euthymic state (91 BD-I, 27 BD-II)<br>76 Healthy controls	64.0±9.7 BD (64.2±9.8 BD-I, 63.3±9.5 BD-II)<br>65.9±9.6 HC	31.29%/68.70% BD (30.7%/69.3% BD-I, 33.3%/66.7% BD-II)<br>48.0%/52.0% HC	Cross-sectional study	SCID-I	ELISA	Serum	Yes	Not specified	Yes
41.	Tatay-Manteiga et al. (2017)	48 BD euthymic patients (25 early-stage, 23 late-stage)<br>23 Healthy siblings of BD patients<br>21 Healthy controls	44.21±10.06 (18-60 years) BD (43.4±10.3 early-stage, 45.1±9.8 late-stage)<br>41.5±11.8 siblings<br>36.7±10.9 HC	47.91%/52.08% BD (48%/52% early-stage, 47.82%/52.17% late-stage)<br>30.43%/69.56% siblings<br>33.33%/66.66% HC	Cross-sectional study	DSM-IV-TR diagnosis criteria	ELISA	Plasma/serum	Yes	Included	Yes
42.	Tunca et al. (2014)	96 BD patients (92 BD-I, 4 BD-II) (37 euthymic, 33 manic, 26 depressed)<br>61 Healthy controls	38.12±10.84 BD (36.24±10.02 euthymia, 36.77±12.02 mania, 42.54±10.52 depression)<br>38.29±11.59 HC	44.74%/55.25% BD (37.8%/62.2% euthymia, 34.3%/65.7% mania, 67.9%/32.1% depression)<br>43.3%/56.7% HC	Cross-sectional study	SCID-I	ELISA	Serum	Medicated and drug-free	Not specified	Yes
43.	Uyanik et al. (2015)	30 BD-I patients in manic episode, with pharmacological treatment during 6 weeks of follow-up<br>28 Healthy controls	33.4±8.6 (18-65 years) BD-I<br>33.1±7.8 HC	53.3%/46.7% BD-I<br>60%/40% HC	Longitudinal study (6 weeks)	DSM-IV-TR diagnosis criteria	ELISA/EDTA	Plasma	Drug free (three groups: 1. never used any treatment; 2. discontinued their treatment for at least 1 month; 3. irregular	Excluded	Yes

									treatment who have not been treated at least one month) at baseline; pharmacological treatment during 6 weeks of follow-up without randomization		
44a.	van den Ameele et al. (2017)	67 BD patients (35 depressive episode [16 BD-I, 19 BD-II], 32 (hypo)manic episode [26 BD-I, 4 BD-II, 2 schizoaffective])<br>30 Healthy controls	43.3±11.1 (23–62 years) BD (43.7±9.7 (28–61 years) depressive, 42.9±12.7 (23–62 years) (hypo)manic)<br>43.0±11.4 (22–61 years) HC	41.8%/58.2% BD (31.4%/68.6% depressive 53.1%/46.9% (hypo)manic)<br>46.7%/53.3% HC	Prospective study	MINI-plus, version 5.0.0	ECLIA	Plasma	Yes	Excluded	Yes
44b.	van den Ameele et al. (2018)	67 BD patients (42 BD-I, 23 BD-II, 2 schizoaffective disorder) (29 depressive episode, 29 (hypo)manic episode, 9 mixed episode)<br>35 Healthy controls	43.3±11.1 (23–62 years) BD<br>42.7±11.6 (23–62 years) HC	41.8%/58.2% BD<br>45.7%/54.3% HC	Cross-sectional study	MINI-plus	ECLIA	Plasma	Yes	Excluded	Yes
45.	Wang et al. (2016)	48 BD-II patients (placebo)<br>41 SBD (subthreshold bipolar disorder: more than 2-days but less than 4-days duration of hypomania) patients (placebo)	31.5±11.3 BD-II<br>28.5±10.2 SBD	54.16%/45.83% BD-II<br>51.21%/48.78% SBP	Prospective and longitudinal study (post-hoc subgroup analysis of a clinical trial)	SADS-L, Chinese version	Antibody pair assay system	Plasma	Yes	Not specified	Yes
46.	Wang et al. (2016)	737 BD patients (234 BD-I, 260 BD-II, 243 SBD)<br>140 Healthy controls	32.69±12.20 BD (33.6±11.7 BD-I, 31.6±12.3 BD-II, 33.0±12.6 SBD)<br>31.9±8.17 HC	47.48%/52.50% BD (49.14%/50.85% BD-I, 51.92%/48.07% BD-II, 41.15%/58.84% SBD)<br>57.85%/42.14% HC	Cross-sectional study	SADS-L, Chinese version	Antibody pair assay system	Plasma	Not specified	Included	Yes
47.	Yoshimura et al. (2006)	18 BD-I patients (12 manic episode, 6 depressive episode), with risperidone treatment during 4 weeks of follow-up<br>20 Healthy controls	34±15 (23-51 years) BD-I<br>30±11 HC	44.44%/55.55% BD-I<br>45%/55% HC	Clinical trial (4 weeks)	DSM-IV diagnosis criteria	ELISA	Plasma	Not specified at baseline; risperidone treatment (co-administered valproic acid or lithium) during 4 weeks of follow-up	Not specified	Not specified

Table S2. Summary of the number of studies included in the systematic review and meta-analysis, separated by biomarker, mood state and symptoms severity.

		Mood State						Symptoms Severity			
**Biomarker**	**Studies included**	**Depression**		**Euthymia**		**Mania**		**Depression Severity (HDRS)**		**Mania Severity (YMRS)**	
		**Number**	**N**	**Number**	**N**	**Number**	**N**	**Number**	**N**	**Number**	**N**
**BDNF**	25	4	147	5	272	6	212	5	271	5	372
**IL-6**	20	1	23	2	141	2	89	4	181	4	202
**TNF-α**	17	3	68	2	141	4	131	2	67	3	180
**IL-10**	8			1	52	1	32	1	48		
**IL-8**	5	1	23	1	89	1	57				
**IL-1B**	4	1	10			1	10				
**sIL-6R**	4	1	14	1	75	1	41	1	130	1	130
**sTNF-R1**	4							1	130	1	130
**IL-4**	3	1	10			1	10				
**sIL-2R**	3	1	14	1	75	1	41	1	130		
**IFN-γ**	3							1	67	2	97
**NGF**	3										
**IL-1Ra**	3										
**IL-18**	3										
**IL-2**	2	1	10			1	10				
**sTNF-R2**	2							1	22	1	22
**TGF-β1**	2										
**sIL-1Ra**	2										
**GDNF**	2										
**NT-3**	2										
**sTNFR60**	2										
**sTNFR80**	2										
**sTNF-6R**	1	1	14	1	75	1	41				
**SVCAM-1**	1	1	44			1	39	1	44	1	83
**SICAM-1**	1							1	44	1	83
**sCD40L**	1										
**sFlt-1**	1										
**IGF-1**	1										
**IL-18BP**	1										
**β-NGF**	1										
**NT-3**	1										
**NT-4/5**	1										
**VEGF**	1										

Table S3. Abbreviations of inflammatory and trophic factors analyzed in the present systematic review and meta-analysis.

Abbreviation	Name
BDNF	brain-derived neurotrophic factor
sCD40L	soluble cluster of differentiation 40 ligand
sFlt-1	soluble fms-like tyrosine kinase 1
GDNF	glial cell line-derived neurotrophic factor
sICAM-1	soluble intercellular adhesion molecule-1
IFN-γ	interferon-gamma
IGF-1	insulin-like growth factor-1
IL-1β	interleukin 1 beta
IL-1RA	interleukin-1 receptor antagonist
sIL-1RA	soluble interleukin-1 receptor antagonist
IL-2	interleukin 2
sIL-2R	soluble interleukin 2 receptor
IL-4	interleukin 4
IL-6	interleukin 6
sIL-6R	soluble interleukin 6 receptor
IL-6RA	interleukin-6 receptor alpha
IL-8	Interleukin 8
IL-10	interleukin 10
IL-18	Interleukin 18
IL-18BP	Interleukin-18 binding protein
NGF	nerve growth factor
β-NGF	beta nerve growth factor
NT-3	neurotrophin-3
NT-4/5	neurotrophin-4/5
TGF-β 1	transforming growth factor beta 1
TNF-α	tumor necrosis factor alpha
sTNFR1	soluble tumor necrosis factor receptor 1
TNFR2	tumor necrosis factor receptor 2
sTNFR2	soluble tumor necrosis factor receptor 2
sTNFR60	soluble tumor necrosis factor receptor 60
sTNFR80	soluble tumor necrosis factor receptor 80
sVCAM-1	soluble vascular cell adhesion molecule-1
VEGF	vascular endothelial growth factor

Annex S1. Details of the search strategy conducted in the present systematic review.

The following key word were used for the search: “bipolar*[Title/Abstract] OR bipolar disorder[Title/Abstract] OR mania*[Title/Abstract] OR manic[Title/Abstract]” AND “inflam* factors[Title/Abstract] OR neuroinflam* factors[Title/Abstract] OR trophic factors[Title/Abstract] OR neurotrophi* factors[Title/Abstract] OR neurotrophins[Title/Abstract] OR growth factors[Title/Abstract] OR cytokin*[Title/Abstract] OR oxidative stress[Title/Abstract] OR neurotoxi*[Title/Abstract] OR neurodevelopment[Title/Abstract] OR neurogenesis[Title/Abstract] OR neuroplasticity[Title/Abstract]” AND “risk factors OR clinic* factors OR social factors OR psychosocial factors OR environment* factors OR physical stress* OR psycholog* stress* OR emotional stress* OR life events OR lifestyle OR epidemiolog* OR demographic factors OR exposure OR triggers factors OR environmental triggers OR vulnerabilit* OR stress* markers OR risks markers OR prenatal factors OR perinatal factors OR maternal exposure OR early adversit* OR childhood maltreatment OR abuse OR trauma OR substance misuse OR substance abuse OR alcohol* OR psychiatric comorbidity OR medical comorbidity OR suicid* OR family history.

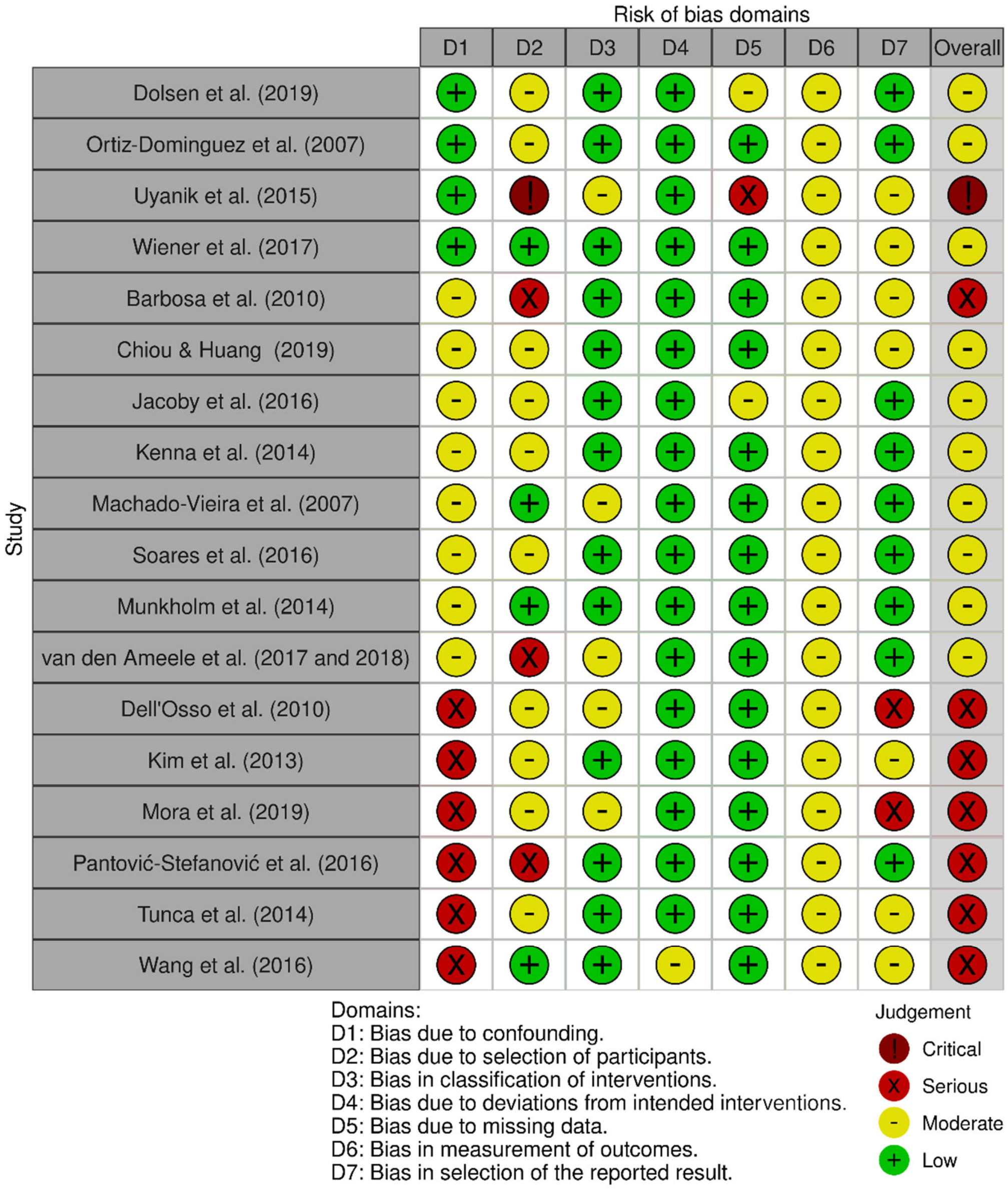

Figure S1. Traffic light plot of the domain-level judgments for risk-of-bias assessment.

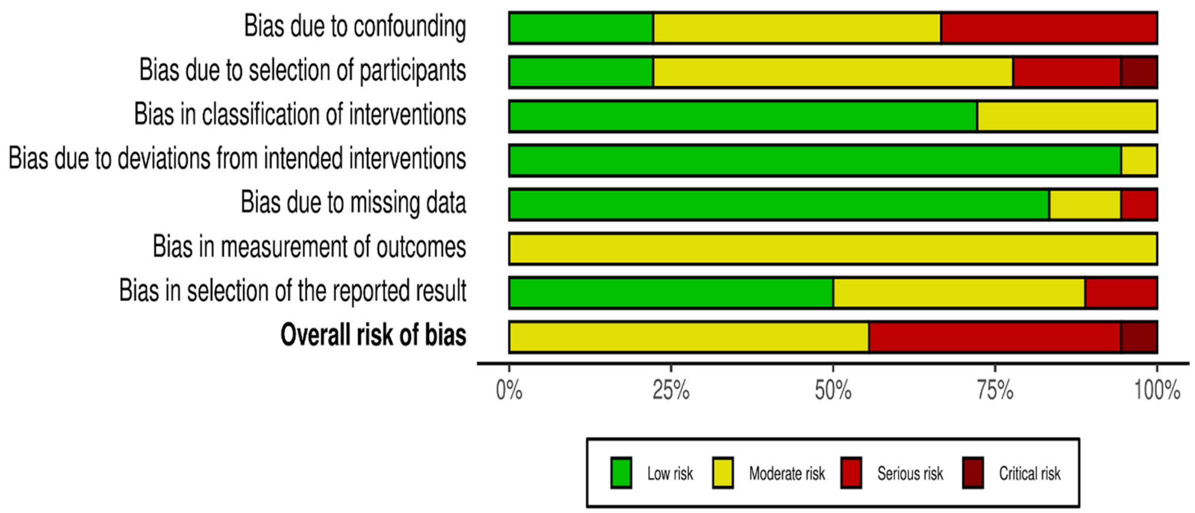

Figure S2. Risk-of-bias summary plot.
